# Early deep sedation is associated with decreased in-hospital and two-year follow-up survival

**DOI:** 10.1186/s13054-015-0929-2

**Published:** 2015-04-28

**Authors:** Felix Balzer, Björn Weiß, Oliver Kumpf, Sascha Treskatsch, Claudia Spies, Klaus-Dieter Wernecke, Alexander Krannich, Marc Kastrup

**Affiliations:** Department of Anaesthesiology and Intensive Care Medicine, Charité - Universitätsmedizin Berlin, Charitéplatz 1, Berlin, 10117 Germany; Charité – Universitätsmedizin Berlin and SOSTANA GmbH, Wildensteiner Straße 27, Berlin, 10318 Germany; Coordination Centre for Clinical Trials, Department of Biostatistics, Charité -Universitätsmedizin Berlin, Charitéplatz 1, Berlin, 10117 Germany

## Abstract

**Introduction:**

There is increasing evidence that deep sedation is detrimental to critically ill patients. The aim of this study was to examine effects of deep sedation during the early period after ICU admission on short- and long-term survival.

**Methods:**

In this observational, matched-pair analysis, patients receiving mechanical ventilation that were admitted to ICUs of a tertiary university hospital in six consecutive years were grouped as either lightly or deeply sedated within the first 48 hours after ICU admission. The Richmond Agitation-Sedation Score (RASS) was used to assess sedation depth (light sedation: −2 to 0; deep: −3 or below). Multivariate Cox regression was conducted to investigate the impact of early deep sedation within the first 48 hours of admission on in-hospital and two-year follow-up survival.

**Results:**

In total, 1,884 patients met inclusion criteria out of which 27.2% (n = 513) were deeply sedated. Deeply sedated patients had longer ventilation times, increased length of stay and higher rates of mortality. Early deep sedation was associated with a hazard ratio of 1.661 (95% CI: 1.074 to 2.567; *P* = 0.022) for in-hospital survival and 1.866 (95% CI: 1.351 to 2.576; *P* <0.001) for two-year follow-up survival.

**Conclusions:**

Early deep sedation during the first 48 hours of intensive care treatment was associated with decreased in-hospital and two-year follow-up survival. Since early deep sedation is a modifiable risk factor, this data shows an urgent need for prospective clinical trials focusing on light sedation in the early phase of ICU treatment.

**Electronic supplementary material:**

The online version of this article (doi:10.1186/s13054-015-0929-2) contains supplementary material, which is available to authorized users.

## Introduction

The management of pain, agitation, stress, discomfort and delirium are important parts of intensive care unit (ICU) therapy. However, due to technical improvements over the past years, deep sedation is no longer required for obtaining tolerance of mechanical ventilation. Several guidelines [1-5] recommend an awake and cooperative patient. Sedation requires a distinct indication, and light levels should be preferred over deep levels of sedation. If invasive procedures are performed or under special conditions such as increased intracranial pressure, sedation may be required for a limited period of time with a well-defined sedation target [1,6-9].

There is increasing evidence that protocols targeting sedation to a level that keeps the patients awake and cooperative can result in shorter ventilation time, shorter ICU stay, lower incidence of delirium and decrease of ICU mortality [10,11]. Despite the availability of these guidelines, surveys have shown that a wide variability exists in the implementation of these recommendations [12-15]. A meta-analysis of sedation studies in the ICU has demonstrated that there is substantial incidence of undesired deep sedation in up to 40% to 60% of all assessments [16].

Prospective trials have shown a negative impact of early deep sedation on patient outcome including delayed extubation, prolonged length of stay (LOS) and increased mortality [17-19]. In order to validate these findings in a European health care system and to account for ‘real-life’ conditions away from study protocols, this study investigated the impact of early deep sedation within the first 48 hours of intensive care by means of a retrospective design. As the term of deep sedation is not clearly defined in the literature, we supplied a statistically robust definition via a two-step approach.

## Methods

This observational analysis was conducted at a university hospital in Berlin, Germany. After written consent of the data authorities and the hospital ethics commission (Ethikkommission der Charité - Universitätsmedizin Berlin, EA1/126/08), clinical routine data were acquired from the two electronic patient data management systems operated at the hospital (COPRA, Sasbachwalden, Germany and SAP, Walldorf, Germany). Due to its retrospective design, the ethics commission waived the need of informed consent for this study. We extracted data from all patients admitted to one of four ICUs of our department between 2007 and 2012. This included two interdisciplinary surgical ICUs, one cardiac ICU that primarily treats patients after cardiac surgery, and one interdisciplinary ICU that is primarily specialised in treating acute respiratory distress syndrome. A neurosurgical ICU also belonging to the department was excluded. The first 48 hours after ICU admission were defined as the study period.

**Figure 1:**
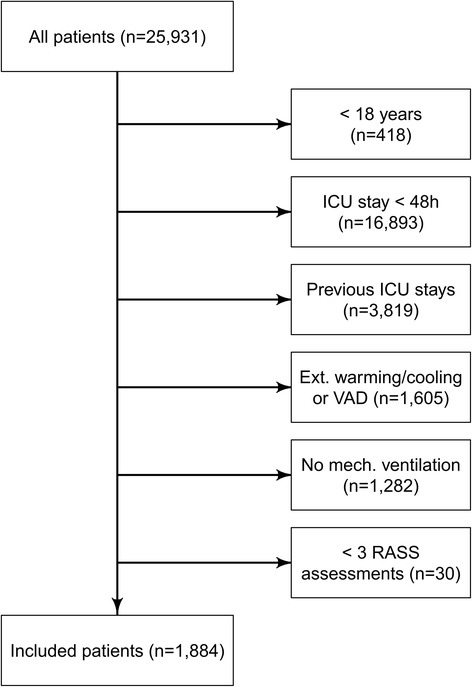
Consolidated Standards of Reporting Trials (CONSORT) diagram.

Patients aged <18 years, LOS in ICU <48 hours, previous stays on one of the department’s ICUs during the same hospital admission, external warming/cooling methods, need for cardiac or pulmonary extracorporeal assist devices and no mechanical ventilation at the time of admission were defined as exclusion criteria (see CONSORT diagram in Figure 1). Furthermore, patients with less than three measurements for 48 hours were excluded from our evaluation to avoid a bias due to an unreliable sedation screening.

At our institution, a nurse-driven monitoring of at least one assessment per shift and established key performance indicators including validated sedation, analgesia, and delirium scores have been implemented [20,21]. Daily Richmond Agitation-Sedation Score (RASS) targets are assessed and documented for all patients by the attending physician. Based on these objectives, sedation is driven by nurses [1].

As the term of deep sedation is not clearly defined in the literature, we aimed to provide a statistically robust definition in this study via a two-step approach. Although there is consensus that a RASS measurement ≤−3 describes a state of deep sedation, it is unclear how this estimation can be applied to a varying number of RASS measurements in an individual patient. In the first step of our preliminary analysis, RASS measurements during the specified time period were converted to single continuous variables by calculating the ratio of RASS measurements ≤−3 and the total number of RASS measurements. This allowed quantification of patients’ degree of deep sedation. Second, we assessed the optimal cutoff for dichotomously distinguishing between deeply and not deeply sedated patients in order to attribute them to two different groups. Therefore, we conducted receiver operating characteristic (ROC) analyses to investigate sensitivity and specificity of the previously calculated sedation variable in regards to the dichotomous outcome parameter of in-hospital mortality. The degree of deep sedation that resulted in the highest sum of sensitivity and specificity qualified as the best cutoff point (Youden index). In the resulting ROC diagram, this value corresponded to the maximal vertical distance between the ROC curve and the diagonal line [22].

Patient age, gender, body mass index, Acute Physiology and Chronic Health Evaluation II (APACHE II) admission score, type of ICU admission (that is medical, emergency surgery, elective surgery), use of inotropics and sedatives during the 48-hour study period were used to characterise the study population. Mortality, LOS, time to extubation, incidence of haemodialysis during the first 48 hours as well as pain and delirium, assessed by the Confusion Assessment Method in the ICU (CAM-ICU) for the whole ICU stay [23] were chosen as outcome parameters. Pain was defined as presence of at least three positive measurements with a numeric rating scale score higher than 4 or a score more than 5 on the behavioural pain scale. Information on long-term mortality, defined as two years after ICU admission, was acquired after clearance from the federal data safety officer by consulting the registry office in Berlin, Germany. Patients with shorter observation intervals, that is less than two years between ICU admission and date of inquiry at the registry office, were excluded in analyses on long-term mortality.

### Statistical analyses

Descriptive analyses and statistical testing were performed using the R Project for Statistical Computing 3.0.1 [24] with a *P* value below 0.05 regarded as significant. When normal distribution was ruled out using the Kolmogorov-Smirnov test, results are given in median and interquartile range (IQR), otherwise mean ± standard deviation (SD). Qualitative observations are characterised by numbers with per cent. Statistical significance among groups is univariately analysed by the exact nonparametric Mann-Whitney *U* test. Exact chi-square tests are used for qualitative data. In order to account for unequal group size and to adjust for significantly different factors among groups that might have affected outcome, deeply sedated patients were matched to control using APACHE II admission score and type of admission as matching criteria prior to multivariate analyses [25]. Survival was analysed using Kaplan-Meier estimations and tested by the log-rank test between groups. Testing multivariately for the impact of early deep sedation on short- and long-term survival, Cox-regression adjusted for patient age, gender, body mass index, APACHE II admission score, type of ICU admission as well as use of inotropic substances and haemodialysis during study period was applied. Sensitivity analyses were conducted addressing the concern whether the long study period (that is six consecutive years) might have had an influence on investigated variables. Therefore, the proportion of deeply sedated patients and defined outcome variables were compared over time. Furthermore, an alternative regression model that included year of admission as explanatory variable was developed in order to investigate its impact on outcome. All tests should be understood as constituting explorative analysis, as no adjustment for multiple testing has been made.

## Results

All patients with complete electronic patient records (n = 25,931) were screened for eligibility. After selection regarding inclusion and exclusion criteria, 1,884 patients were included in the final analysis (Figure 1). Data regarding long-term mortality was available for 1,457 patients from which 139 had to be excluded since their observation period was smaller than the previously defined interval of two years.

In order to set the optimal cutoff point for dichotomously distinguishing between deeply and lightly sedated patients, we selected the degree of deep sedation that maximised the vertical distance between the ROC curve and diagonal line (highest sum of sensitivity and specificity) [22]. Sensitivity analyses for distinguishing between deeply and not deeply sedated patients resulted in a cutoff point of 85%. Hence, patients who had more than 85% of documented RASS scores during the study period equal or below −3 were attributed to the deeply sedated group (group DS; n = 513). The remaining patients were labelled as not deeply sedated (group NDS; n = 1,371). Consequently, deeply sedated patients had a lower first RASS assessment (−5 [−5; -4] vs. −4 [−5; -1]; *P* <0.001) and reached a less sedated state defined as RASS >−3 at a later point of time (78 h [52;141] vs. 11 h [5;20]; *P* <0.001). Table 1 gives the summary of descriptive data for the patient population including used drugs for sedation.

**Table 1:** **Basic patient characteristics for unmatched (left) and matched (right) patient population**

	**Unmatched**			**Matched (APACHE II and type of admission)**		
	**Not deeply sedated (n = 1371)**	**Deeply sedated (n = 513)**	***P***	**Not deeply sedated (n = 510)**	**Deeply sedated (n = 510)**	***P***
Age [y]	68 [59;75]	64 [50;73]	<0.001	68 [58;75]	64 [50;72]	<0.001
Male gender	881 (64.3%)	363 (70.8%)	0.009	319 (62.5%)	361 (70.8%)	0.006
Body mass index	26.2 [23.6;30.7]	27.1 [23.5;31.0]	0.176	26.2 [23.8;30.5]	27.1 [23.5;31.0]	0.270
APACHE II on ICU admission	20 [16;26]	25 [18;31]	<0.001	25 [18;30]	25 [18;31]	0.552
Type of admission:			<0.001			<0.952
• Elective surgery	697 (53.2%)	136 (27.6%)		142 (28.5%)	136 (27.6%)	
• Emergency surgery	348 (26.5%)	170 (34.5%)		169 (33.9%)	170 (34.5%)	
• Medical	266 (20.3%)	187 (37.9%)		188 (37.7%)	187 (37.9%)	
Inotropics			<0.001			<0.001
• None	267 (20.4%)	29 (5.73%)		102 (20.7%)	29 (5.73%)	
• Dopamine ≤5	176 (13.5%)	6 (1.19%)		42 (8.52%)	6 (1.19%)	
• Dopamine >5 or E/NE ≤0.1	541 (41.4%)	115 (22.7%)		212 (43.0%)	115 (22.7%)	
• Dopamine >15 or E/NE >0.1	324 (24.8%)	356 (70.4%)		137 (27.8%)	356 (70.4%)	
Sedatives			<0.001			<0.001
• Propofol	1048 (76.4%)	191 (37.2%)		372 (72.9%)	189 (37.1%)	
• Midazolam	26 (1.9%)	99 (19.3%)		15 (2.94%)	99 (19.4%)	
• Both	129 (9.4%)	211 (41.1%)		62 (12.2%)	210 (41.2%)	
• None of above	168 (12.3%)	12 (2.34%)		61 (12.0%)	12 (2.35%)	
First RASS	−4 [−5;-1]	−5 [−5;-4]	<0.001	−4 [−5;-1]	−5 [−5;-4]	<0.001
Time till first RASS [h]	1 [0;4]	2 [1;4]	0.155	1 [0;4]	2 [1;4]	0.051
Time till first RASS >−3 [h]	11 [5;20]	78 [52;141]	<0.001	11 [4;21]	79 [52;141]	<0.001

APACHE II, Acute Physiology and Chronic Health Evaluation; ICU, intensive care unit; E, epinephrine; NE, norepinephrine; RASS, Richmond Agitation-Sedation Score.

Patients were matched based on APACHE II admission score and type of admission. Out of 513 deeply sedated patients, 510 patients were able to be matched to a not deeply sedated counterpart. Significant differences were observed regarding the following characteristics: Patients of the deeply sedated group were significantly younger (64 [50;72] vs. 68 [58;75]; *P* <0.001), inotropic drugs were used in higher dosages, and benzodiazepines were more frequently used.

**Figure 2:**
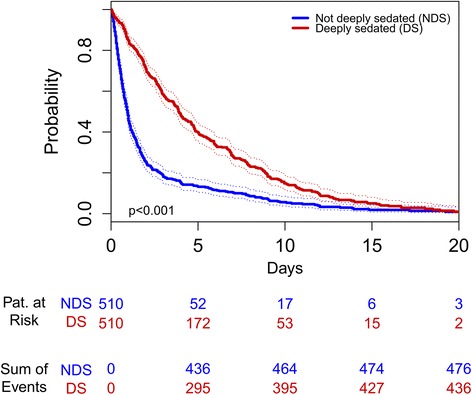
Kaplan-Meier curves for time to extubation (hours from admission to the ICU until extubation of the patient) in matched cohort. NDS, not deeply sedated; DS, deeply sedated.

**Figure 3:**
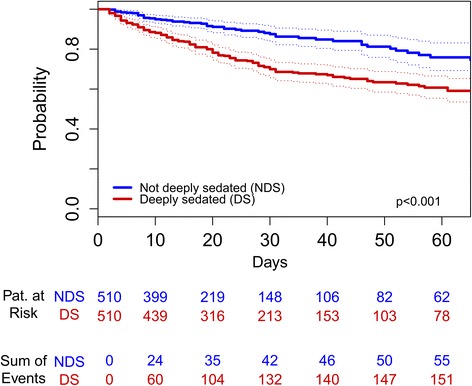
Kaplan-Meier curve for in-hospital survival (all causes) in matched cohort. NDS, not deeply sedated; DS, deeply sedated.

**Figure 4:**
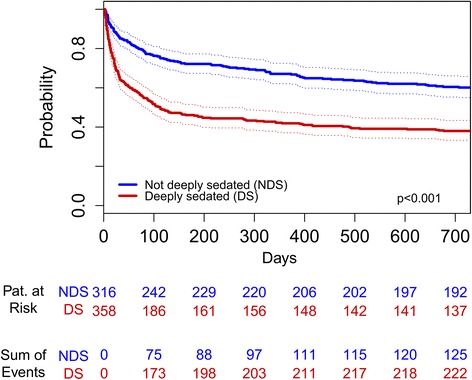
Kaplan-Meier curves for the two-year survival in matched cohort. NDS, not deeply sedated; DS, deeply sedated.

In both unmatched and matched population, deeply sedated patients showed an increased mortality (ICU, in-hospital, and long-term), longer LOS (ICU, in-hospital), longer time to extubation and more frequently need of renal replacement therapy during first 48 hours after ICU admission (Table 2). Measured pain was lower in deeply sedated patients (8.2% vs. 24.5%, *P* <0.001 in matched population). Higher incidence of delirium in deeply sedated patients was seen in the unmatched population, but differences were no longer significant after matching on APACHE II admission score. Kaplan-Meier curves for time to extubation, in-hospital and long-term survival are shown in Figures 2, 3, and 4 (log-rank test of *P* <0.001 in all figures for group differences).

**Table 2:** **Outcome parameters for unmatched (left) and matched (right) patient population**

	**Unmatched**			**Matched (APACHE II and type of admission)**		
	**Not deeply sedated (n = 1,371)**	**Deeply sedated (n = 513)**	***P***	**Not deeply sedated (n = 510)**	**Deeply sedated (n = 510)**	***P***
Mortality (ICU)	67 (4.89%)	137 (26.7%)	<0.001	37 (7.25%)	137 (26.9%)	<0.001
Mortality (hospital)	131 (9.56%)	175 (34.1%)	<0.001	67 (13.1%)	175 (34.3%)	<0.001
Mortality (2 years)	307 (32.0%)	222 (61.8%)	<0.001	126 (39.9%)	222 (62.0%)	<0.001
LOS (ICU) [d]	8 [5;16]	21 [12;38]	<0.001	10 [6;23]	21 [12;38]	<0.001
LOS (hospital) [d]	18 [12;33]	28 [16;48]	<0.001	19 [11;38]	28 [16;48]	<0.001
Time to extubation [h]	17 [8;33]	75 [37;156]	<0.001	21 [10;41]	76 [37;160]	<0.001
Delirium	445 (32.5%)	216 (42.1%)	<0.001	213 (41.8%)	216 (42.4%)	0.899
Haemodialysis during first 48 hours	153 (11.2%)	204 (39.8%)	<0.001	73 (14.3%)	204 (40.0%)	<0.001
NRS ≥5 or BPS ≥6 during first 48 hours	342 (24.9%)	42 (8.2%)	<0.001	125 (24.5%)	42 (8.2%)	<0.001

ICU, intensive care unit; LOS, length of stay; NRS, numeric rating scale; BPS, behavioural pain scale.

In order to account for remaining confounders after pair matching, we conducted Cox regression for both short-term (that is in-hospital) and long-term (that is two-year follow-up), adjusting for APACHE II, age, gender, body mass index, type of ICU admission, inotropics and sedatives. Deep sedation was associated with an approximately two-fold risk of dying in both short-term survival (hazard ratio (HR) = 1.661; 95% confidence interval (CI) 1.074 to 2.567; *P* = 0.022) and long-term survival (HR = 1.866; 95% CI 1.351 to 2.576; *P* <0.001). Besides deep sedation, APACHE II admission score remained significant in both regression models (Table 3).

**Table 3:** **Cox regression analysis for in-hospital survival (left) and two-year survival (right)**

	**Cox regression for in-hospital survival**			**Cox regression for two-year follow-up survival**		
	***P***	**HR**	**95% CI**	***P***	**HR**	**95% CI**
Deep sedation during first 48 h on ICU	0.022	1.661	1.074-2.567	<0.001	1.866	1.351-2.576
APACHE II on ICU admission	<0.001	1.051	1.030-1.073	<0.001	1.045	1.028-1.062
Age [y]	0.072	1.011	0.999-1.022	0.001	1.016	1.007-1.026
Male gender	0.132	0.764	0.538-1.084	0.024	1.375	1.043-1.812
Body mass index	0.043	0.973	0.947-0.999	0.064	0.978	0.955-1.001
Admission: emergency surgery	0.398	0.408	0.051-3.264	0.960	1.021	0.449-2.321
Admission: medical	0.421	1.355	0.647-2.837	0.950	1.016	0.626-1.647
Inotropics: dopamine ≤=5	0.226	1.538	0.766-3.090	0.970	0.991	0.614-1.600
Inotropics: dopamine >5 or E/NE ≤=0.1	0.102	1.473	0.926-2.344	0.764	0.948	0.670-1.341
Inotropics: dopamine >15 or E/NE >0.1	0.321	1.270	0.792-2.037	0.041	1.441	1.015-2.046
Sedatives: propofol	0.877	0.972	0.675-1.399	0.189	1.224	0.905-1.654
Sedatives: midazolam	0.618	1.272	0.494-3.277	0.497	0.823	0.469-1.444
Sedatives: both	0.265	1.779	0.646-4.902	0.326	1.388	0.722-2.667
Haemodialysis during first 48 h on ICU	0.416	1.502	0.564-3.998	0.600	0.850	0.464-1.560

Regression analysis of factors influencing in-hospital and long-term mortality in matched cohort. Reference category for type of admission: elective surgery, for inotropics: neither dopamine, epinephrine nor epinephrine, for sedatives: none. HR, hazard ratio; CI, confidence interval; APACHE II, Acute Physiology and Chronic Health Evaluation; ICU, intensive care unit; E, epinephrine; NE, norepinephrine.

Results of sensitivity analyses regarding the influence of the long study period revealed that the number of deeply sedated patients remained constant and that defined study end points remained considerably stable over time. During the last five years of the study, the proportion of deeply sedated patients ranged from 27.2% to 32.4% (*P* = 0.657). In-hospital mortality decreased over time (17.8% in 2008 to 10.3% in 2012), however these changes were not statistically significant (*P* = 0.057). Long-term mortality decreased from 39.9% in 2008 to 34.9% in 2012 (*P* = 0.008). LOS in the ICU and time to extubation remained statistically unchanged over time (*P* = 0.755 and *P* = 0.982, respectively), see Table S1 in Additional file 1. When the year of admission was included in an alternative Cox regression model on short- and long-term survival, HRs for early deep sedation remained essentially stable (short-term survival: 1.661, 95% CI 1.074 to 2.567, *P* = 0.022 to 1.685, 95% CI 1.094 to 2.597, *P* = 0.018; long-term survival: 1.866, 95% CI 1.351 to 2.576, *P* <0.001 to 1.846, 95% CI 1.339 to 2.545, *P* <0.001), see Table S2 in Additional file 1.

## Discussion

In this observational, matched-pairs analysis, we demonstrated that early deep sedation impairs clinical outcomes as it leads to longer mechanical ventilation and prolonged LOS. In multivariate analyses, early deep sedation was associated with both decreased in-hospital and two-year follow-up survival. These findings are consistent with recently published trials in other health care systems. The Australian New Zealand sedation practice in intensive care evaluation (ANZ SPICE) study included 251 patients and demonstrated that depth of early sedation predicted delayed extubation and increased in-hospital and 180-day mortality [26]. Results of this study were replicated in a prospective longitudinal multicentre cohort study in Malaysian ICUs including 259 patients from 11 hospitals [19]. Similar, in a secondary analysis of a multicentre prospective cohort conducted in 45 Brazilian ICUs, early deep sedation was associated with adverse outcomes including mortality [27]. In the latter study, the Glasgow Coma Scale was used as a surrogate for sedation depth and yielded an increased risk for in-hospital mortality in deeply sedated patients.

In comparison to previous studies, our analysis comprises several novel aspects: First, we propose a definition of deep sedation in order to contribute to the comparability of studies. The term of ‘over-sedation’ is not clearly defined in the literature as a systematic review from 2008 by Jackson *et al*. demonstrated a wide variation of its definition [16]. In our study, we conducted ROC analyses regarding the proportion of deep sedation (that is RASS measurements ≤−3 and the total number of RASS measurements) and in-hospital mortality. This way we accounted for a varying number of RASS measurements in a defined study period. The resulting Youden index of 85% presented the best cutoff for distinguishing between deeply and not deeply sedated patients. In consequence, the duration of deep sedation seems to be relevant, which supports the practice of sedative interruptions [9]. Second, the setting of our study was different from previous analyses. Our analysis was carried out in a different health care system by using clinical routine data from a major German university hospital, including a relatively high number of patients. This approach was intended to reflect ‘real-life’ conditions from a European tertiary medical centre in order to validate previous findings. Furthermore, the retrospective study design allowed inclusion of eligible patients right after admission to the ICU. Therefore, we considered the depth of sedation from the very beginning of treatment whereas prospective studies usually imply a certain delay due to the screening and inclusion process.

Our institutional goal is to measure RASS scores at least every 8 hours, but more frequently if necessary. Assuming that score assessment is intensified during arousal periods or change of target RASS, one has to assume a nearly stable course of RASS between measurements. The time spent deeply sedated would count if the assumption was true. This should be investigated by prospective trials. In this study, we did not address the question of compliance with our institution’s standard operating procedures regarding sedation [28]. However, previous research had yielded strong adherence to quality indicators for sedation at our institution [29].

Overall it appears that during the very early ICU period, patients are sedated deeper than during late phases of treatment. There are no published data explaining this comprehensively. One possible explanation could be haemodynamic instability since the deeply sedated group received higher amounts of vasopressors and inotropics. Of course, this could also be a consequence of the deep sedation as cause-effect chains cannot be proven in retrospective designs. The argument that an increased severity of illness is the cause for deep sedation could be advanced in this context. Even patients with acute lung injury, usually particularly at risk for deep sedation, could be treated with less sedatives and kept awake without signs of delirium in a monocentric randomised controlled trial performed by Hager and colleagues [30]. Other studies targeting light sedation that included patients from the beginning of their ICU stay also included patients independent of the severity of disease [17]. Whether the sedative itself has an effect on outcome requires further investigation. A recent study suggested that patients receiving propofol had a reduced risk of mortality as well as an increased likelihood of earlier ICU discharge and earlier discontinuation of mechanical ventilation compared to a treatment with benzodiazepines [31].

### Limitations

There are several limitations that have to be considered in the interpretation of our results. First, deep sedation may have been introduced for medical reasons, thus limiting generalisability of findings. However, this aspect was militated as much as possible by excluding patients that were bound for these indications. A neurosurgical ICU of our centre was not considered eligible, and we excluded all patients receiving any form of external warming or cooling (for example patients in need for cooling after cardiopulmonary resuscitation). Also, patients requiring extracorporeal assist devices were not included in analyses. Further indications for sedation like haemodynamic instability were considered in regression analyses. Second, the quality of data that was considered in this retrospective analysis may have been inferior to that of prospective trials. On the other hand, we think that the use of retrospective data contributes to creating a realistic picture of clinical routine. Such analysis may be essential to validate findings of prospective trials. Lastly, inclusion of patients from a six-year time period might suggest an influence on results due to changes of sedation practices and ICU treatments. However, standard operating procedures for sedation were introduced before the start of the study period. Hence, we were able to rule out an impact due to potential changes of sedation practice [20,21]. In sensitivity analyses, the proportion of deeply sedated patients remained constant over the entire period. Including patient’s admission year in an alternative multivariate regression analyses did not significantly affect the previously calculated hazard ratios.

## Conclusions

Early deep sedation in the first 48 hours of intensive care treatment is associated with increased short- and long-term mortality. To our best of knowledge, this is the first European study that confirms previous findings of prospective trials and the first that demonstrates it in clinical routine worldwide. The study has an impact on the design of further prospective trials comparing sedatives and gives a clear definition for deep sedation.

## Key messages

- Early deep sedation is a predictor for short- and long-term mortality in clinical routine.
- Early deep sedation can be defined as more than 85% of RASS values ≤−3 during the first 48 hours of ICU treatment.
- Although a sedation protocol had been implemented in the study centre since 2006, the incidence of early deep sedation was approximately 30%.

## Additional file

Additional file 1:
**Electronic supplement. **Tables S1 and S2, which contain results of statistical analyses concerning the robustness of the presented data.

## Abbreviations

APACHE II: Acute Physiology and Chronic Health Evaluation II
CAM- ICU: Confusion Assessment Method
CI: confidence interval
HR: hazard ratio
ICU: intensive care unit
IQR: interquartile range
LOS: length of stay
RASS: Richmond Agitation-Sedation Score
ROC: receiver operating characteristics
SD: standard deviation
