# Supplementary material for: Early deep sedation is associated with decreased in-hospital and two-year follow-up survival
Source: Crit Care. 2015 Apr 28;19(1):197. doi: 10.1186/s13054-015-0929-2 (PMC4435917; doi:10.1186/s13054-015-0929-2)
Supplement: Additional file 1: — Electronic supplement. Tables S1 and S2, which contain results of statistical analyses concerning the robustness of the presented data. [file 13054_2015_929_MOESM1_ESM.docx]

Table S1: Number of deeply sedated patients and outcome parameters in their course over the five last years of the study period.

Table S2: Alternative regression model including year of admission as additional variable in order to analyse its impact on early deep sedation on both short- (left) and long-term survival (right). Compared to the original regression model, hazard ratios for early deep sedation remained essentially stable (short-term survival: 1.661, 95% CI 1.074-2.567, p=0.022 to 1.685, 95% CI 1.094-2.597, p=0.018; long-term survival: 1.866, 95% CI 1.351-2.576, p<0.001 to 1.846, 95% CI 1.339-2.545, p<0.001).
